# Energetic and informational masking place dissociable demands on listening effort: Evidence from simultaneous electroencephalography and pupillometrya)

**DOI:** 10.1121/10.0020539

**Published:** 2023-08-23

**Authors:** Sarah Villard, Tyler K. Perrachione, Sung-Joo Lim, Ayesha Alam, Gerald Kidd

**Affiliations:** Department of Speech, Language, and Hearing Sciences, Boston University, Boston, Massachusetts 02215, USA

## Abstract

The task of processing speech masked by concurrent speech/noise can pose a substantial challenge to listeners. However, performance on such tasks may not directly reflect the amount of *listening effort* they elicit. Changes in pupil size and neural oscillatory power in the alpha range (8–12 Hz) are prominent neurophysiological signals known to reflect listening effort; however, measurements obtained through these two approaches are rarely correlated, suggesting that they may respond differently depending on the specific cognitive demands (and, by extension, the specific type of effort) elicited by specific tasks. This study aimed to compare changes in pupil size and alpha power elicited by different types of auditory maskers (highly confusable intelligible speech maskers, speech-envelope-modulated speech-shaped noise, and unmodulated speech-shaped noise maskers) in young, normal-hearing listeners. Within each condition, the target-to-masker ratio was set at the participant's individually estimated 75% correct point on the psychometric function. The speech masking condition elicited a significantly greater increase in pupil size than either of the noise masking conditions, whereas the unmodulated noise masking condition elicited a significantly greater increase in alpha oscillatory power than the speech masking condition, suggesting that the effort needed to solve these respective tasks may have different neural origins.

## I. INTRODUCTION

A growing body of research has demonstrated that the degree of cognitive resources expended by a listener when attending to and processing target speech, often termed “listening effort,” is dissociable from their performance on speech intelligibility tasks (e.g., Koelewijn *et al.*, 2012a; Winn and Teece, 2021). Assessing listening effort—particularly under adverse conditions—may therefore provide valuable insight into the experience of the listener that is not available when measuring speech intelligibility alone (Peelle, 2018; Rennies *et al.*, 2014; Rennies *et al.*, 2019; Winn and Teece, 2021; Zekveld *et al.*, 2010). Quantifying the amount of listening effort expended during a given task allows us to move beyond the standard question, “Under what conditions can the listener solve this task?” to further ask, “What does solving this task demand of the listener?” Addressing this second question adds another dimension to the study of auditory masking, degradation, and other manipulations of speech stimuli that place challenging cognitive demands on the listener.

One area where the examination of listening effort may be of particularly high practical importance is in the context of auditory masking. A great deal of typical human conversation takes place in the presence of extraneous background speech and/or other sources of “noise” that must be filtered out by the listener, a dilemma often referred to as the “cocktail party problem” (Cherry, 1953). Determining the role of listening effort in solving cocktail party-like problems may lead to a better understanding of not only how breakdowns in processing and comprehension in adverse listening situations occur, but also how to mitigate them. There are two functionally distinct sources of auditory masking that likely have different effects on listening effort: *Energetic masking* (EM) is the result of spectrotemporal overlap between target and masker energy and is thought to result from peripheral auditory mechanisms, whereas *informational masking* (IM) consists of additional masking that cannot be explained by spectrotemporal overlap and is believed to result from breakdowns in central processing (Kidd *et al.*, 2008). Most masking conditions involve some degree of both EM and IM. However, it is generally the case that noise masking conditions produce primarily EM, whereas speech masking conditions—particularly when the masking speech is intelligible—produce higher amounts of IM. The IM that is present when speech masks other speech results in part from confusions between target and masker sources on the part of the listener, despite adequate audibility of the target [see Kidd and Colburn (2017) for a review of IM under speech masking conditions].

A key question in examining listening effort under auditory masking conditions, therefore, is to assess how it is affected by EM vs IM. Answering this question would add to the current understanding of the nature of how EM and IM are experienced and processed by listeners in everyday communicative situations. However, this goal is complicated by the absence (thus far) of a gold standard approach to measuring listening effort. Listening effort has sometimes been assessed through self-report (e.g., Rennies *et al.*, 2019); however, two candidate neurophysiological signatures are also thought to quantitatively index listening effort: increases in pupil dilation, as measured by pupillometry, and increases in neural oscillatory power in the alpha frequency range (8–12 Hz), as measured by electroencephalography (EEG). Pupillometry is at this point an established method of measuring listening effort (Koelewijn *et al.*, 2012a; Winn, 2018; Zekveld *et al.*, 2010, 2018). Whereas the discussion about the precise nature and role of the alpha power element of the EEG signal is still ongoing, it has been observed to change as a function of task difficulty and has increasingly been used to measure effort (Obleser *et al.*, 2012; Sauseng and Klimesch, 2008), often alongside pupillometry (Alhanbali *et al.*, 2019; Haro *et al.*, 2022; Fiedler *et al.*, 2021; McMahon *et al.*, 2016; Miles *et al.*, 2017; Seifi Ala *et al.*, 2020). Using neurophysiological approaches to assess listening effort is advantageous in that these approaches do not rely on the listener to subjectively scale or report their own effort.

Interestingly, studies that have assessed listening effort using both pupil dilation and alpha power have typically found that measurements obtained via these two tools are not correlated with one another (e.g., Alhanbali *et al.*, 2019; McMahon *et al.*, 2016; Miles *et al.*, 2017; Seifi Ala *et al.*, 2020). This lack of correlation—along with the fact that pupil size and alpha power have different neural origins—suggests that listening effort may be a multifaceted construct involving processes with two or more distinct neural origins assayed independently by these techniques (Alhanbali *et al.*, 2019; Visentin *et al.*, 2022). Pupil dilation driven by cognitive (as opposed to sensory) processes reflects neural activity in the subcortical locus coeruleus-norepinephrine system [Gilzenrat *et al.*, 2010; Joshi *et al.*, 2016; see van der Wel and van Steenbergen (2018) for a review]. In contrast, alpha power reflects synchronous oscillations of cortical neurons and has been shown to increase during listening tasks involving acoustic degradation and/or high working memory load (e.g., Obleser *et al.*, 2012; Petersen *et al.*, 2015; Tuladhar *et al.*, 2007; Wöstmann *et al.*, 2017), presumably due to the increased cognitive resources required to solve these tasks. However, it remains unknown which facet(s) of listening effort is captured by the neuromodulatory systems that affect pupil dilation vs those that are reflected in changes in the neural alpha oscillatory power.

The goal of the current study was to use both pupillometry and EEG to compare the amount of listening effort exerted under carefully controlled high-IM vs high-EM listening conditions. A central underlying question in this work was whether pupil size and alpha power may respond differently to the listening effort required to solve tasks with high-EM vs high-IM demands. Whereas a number of previous studies have used pupillometry to compare the effects of noise masking and speech masking conditions on listening effort, some results have suggested that high-IM listening conditions elicit a greater pupil response (indicating a higher degree of listening effort) than high-EM listening conditions (e.g., Koelewijn *et al.*, 2012a; Wendt *et al.*, 2018), whereas others have found EM and IM to elicit comparable pupil responses (e.g., Ohlenforst *et al.*, 2018, Versfeld *et al.*, 2021). In terms of EEG findings, most studies that have examined the effect of masking on listening effort as measured by alpha power have tended to focus specifically on high-EM speech-in-noise tasks (e.g., Alhanbali *et al.*, 2019; Dimitrijevic *et al.*, 2017, 2019; McMahon *et al.*, 2016; Miles *et al.*, 2017; Wisniewski *et al.*, 2021). We are not aware of any listening effort studies to date that have directly compared the effect of EM vs IM on alpha power.

In designing the current study, several key considerations were taken into account. First, to obtain measurements of listening effort that could be appropriately compared across participants and conditions, we implemented an approach that held performance (i.e., accuracy) at a constant level across participants and conditions. By keeping behavioral performance constant, differences in the psychophysiological dependent measures across conditions would presumably reflect differences in the amount of effort needed to obtain a particular level of performance. A second consideration was the selection of target and masker stimuli, especially the speech masker stimuli used to create a high-IM listening condition. In prior studies employing speech-on-speech masking conditions, speech maskers have typically consisted of materials such as a portion of a string of concatenated sentences (Koelewijn *et al.*, 2012a; Ohlenforst *et al.*, 2018; Versfeld *et al.*, 2021) or one or more talkers reading the newspaper (Wendt *et al.*, 2018). Although these maskers surely produced some IM, they likely were fairly easy to distinguish from the short target sentences in the majority of trials, due to the multiple source-segregation cues available, such as differences in intonation, syntactic structure, topic, rate of speech, relative stimulus onsets/offsets, etc. [for a review, see Mattys *et al.* (2012); Kidd and Colburn (2017)], as well as the impossibility of explicit target-masker confusions [which is a way of gauging the amount of IM; see Brungart *et al.* (2001) and Kidd *et al.* (2016)]. Thus, it is unclear whether the stimuli and tasks employed in these studies created high uncertainty (and, thus, high IM) in the listener. Several other studies examining the effects of masking on listening have utilized vocoded target and masker sentences (Obleser and Weisz, 2012; Obleser *et al.*, 2012; McMahon *et al.*, 2016, Miles *et al.*, 2017), which allowed for examination of the effects of degrading the speech stimuli but did not allow for clear measurements of the effects of EM or IM, as vocoding affects the amount of EM due to (presumably) increased target-masker overlap within corresponding channels.

With this existing literature in mind, the current study aimed to compare the effects of EM and IM as directly as possible through the utilization of matrix-style sentences. This approach helped to maximize confusability between target and masker, thereby increasing the amount of IM, and provided the opportunity for the analysis of explicit target-masker confusions. Finally, the current study also implemented spatial separation between target and maskers across conditions to allow listeners the opportunity to clearly locate the different talkers by taking advantage of binaural/spatial cues. This experimental design—combining matrix-style sentences, same-gender target and masker talkers, and spatial separation—was expected to produce significant IM with plausible source locations and is one that our lab has used in previous studies with the same stimuli (e.g., Kidd *et al.*, 2016).

The possible outcomes of the study were as follows: If changes in pupil size and alpha oscillatory power both reflect listening effort (broadly defined), we would expect both measures to exhibit a similar pattern of changes for high-EM and high-IM auditory maskers. In contrast, if these two neurophysiological indices reflect different facets of the complex construct of listening effort, we would expect the two measures to exhibit distinct patterns of changes for high-EM and high-IM auditory maskers. We anticipated the second outcome to be more likely for several reasons. First, EM and IM are thought to result from breakdowns in different stages of processing and, therefore, present different task demands to the listener. It is possible that the neural processes that are responsive to these differing task demands are recruited disproportionately by high-IM vs high-EM listening situations. Second, previous studies have reported no correlations between EEG and pupillometry results; indeed, in some cases, results have even been shown to trend in opposite directions (e.g., Miles *et al.*, 2017). Specifically, based on the literature and our own preliminary observations, we hypothesized that a high-IM condition would result in greater changes in pupil size than the high-EM conditions, whereas the high-EM conditions would result in greater changes in alpha power than the high-IM condition.

## II. METHODS

### A. Participants

Young, normal-hearing adult listeners participated in this experiment (*N* = 15; 10 female, 5 male; 6 Asian, 1 Black, 2 multiracial, 6 white;^1^ mean age = 20.8, range = 18–24). Participants were recruited through the existing database of the Psychoacoustics Laboratory at Boston University, as well as through online postings at Boston University. All participants underwent a pure tone audiometric hearing screening to confirm normal hearing, defined here as thresholds of 20 dB hearing level (HL) or better at octave frequencies from 0.25 to 8 kHz in both the left and right ears. All participants reported that they were native English speakers with no diagnosis of attention deficit disorder or history of head injury resulting in loss of consciousness.^2^

### B. Experimental stimuli

Auditory stimuli consisted of recordings of 48 words, each recorded in isolation by eight adult female talkers (Table I). These included 40 one-syllable words drawn from a corpus that has been used in a number of previous studies in our laboratory (e.g., Kidd *et al.*, 2016) plus eight two-syllable names drawn from a separate corpus recorded by the same talkers. For the listening conditions involving unintelligible noise maskers, speech spectrum-shaped noise was created using the long-term average of randomly chosen segments of the entire corpus of recordings used in the study. Two noise conditions were included: In one noise condition, the noise was modulated using the broadband temporal envelopes drawn from words in the corpus, and in the other, the noise was left unmodulated. This approach allowed for the examination of any possible effect of the speech envelope on listening effort.

**TABLE I.** List of all words used in the experiment.

Names	Verbs	Numbers	Adjectives	Objects	Additional two-syllable names
Bob	bought	two	big	bags	Allen
Jane	found	three	cheap	cards	Doris
Jill	gave	four	green	gloves	Kathy
Lynn	held	five	hot	hats	Lucy
Mike	lost	six	new	pens	Peter
Pat	saw	eight	old	shoes	Rachel
Sam	sold	nine	red	socks	Thomas
Sue	took	ten	small	toys	William

Visual stimuli consisted of columns of typed words presented as response options, arranged vertically on a graphical user interface (GUI) on a computer screen. All stimuli were presented using custom scripts in matlab (MathWorks, Inc., Natick, MA) and Psychtoolbox.

### C. Experimental setup

**FIG. 1.**
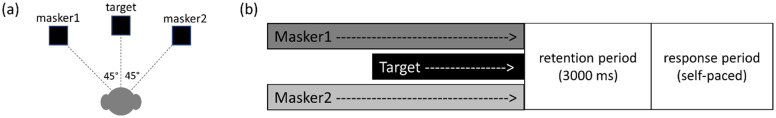
(Color online) (a) Spatial presentation of target and maskers across all conditions. (b) Trial structure across all conditions.

The experiment consisted of a series of adaptive tracking blocks, followed by a pupillometry/EEG recording session. During both portions of the experiment, participants were seated in front of a computer monitor in a dimly lit, sound-attenuated booth. Stimuli were presented through three loudspeakers, each located approximately 1.5 m from the listener's head and positioned at 0° and ±45° azimuth in the horizontal plane. The computer monitor was also located at 0° azimuth, directly in the participant's line of sight; because of this, the loudspeakers were each raised approximately 12 in. above the participant's line of sight in the vertical plane. Target stimuli were always presented from the loudspeaker located at 0° azimuth. Maskers, when present, were always presented simultaneously from the two loudspeakers located at ±45° azimuth [see Fig. 1(a)].

### D. Trial structure

Across the entire experiment, target stimuli consisted of single-word recordings concatenated into five-word sentences. The target sentence always began with the designated target cue word “Sue,” followed by a one-syllable verb, number, adjective, and noun, with each of the latter four words randomly selected from the list of eight possibilities in that category. Examples of possible target sentences include “Sue sold eight red toys” and “Sue found three old gloves.” The target words on each trial were all spoken by one consistent talker, but which talker (among the eight) varied randomly across trials.

**FIG. 2.**
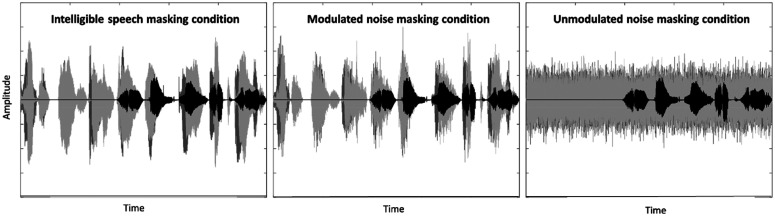
Sample waveforms in each masking condition. Target is represented in black; two maskers are represented in light and dark gray.

Across all masking conditions, onset of the two simultaneous maskers always preceded onset of the target sentence [see Fig. 1(b) for a depiction of the trial structure]. In the intelligible speech masking condition, each masker consisted of an eight-word string, beginning with three randomly drawn two-syllable names, followed by a five-word sentence drawn from the same matrix as the target sentences. Examples of possible masker strings include “William Peter Kathy Bob lost ten green socks” and “Allen Rachel Lucy Pat bought six small hats.” The last five words of the masker strings were always presented simultaneously with the five-word target sentence, with the onsets of each triad of words (e.g., target verb, Masker1 verb, and Masker2 verb) aligned. Like the target word recordings, the words in each masker stream were all spoken by a unique, consistent talker, but the particular talkers in the maskers varied randomly across trials. Within a given trial, the words chosen for Masker1, Masker2, and the target sentence were selected randomly without replacement and, thus, were mutually exclusive; similarly, the Masker1 talker, Masker2 talker, and target talker also were mutually exclusive random selections within a given trial. In the unmodulated noise masking condition, each string of eight masker words was replaced by a single continuous token of speech spectrum-shaped noise with the same duration as the eight-word string. In the modulated noise masking condition, each masker consisted of a similar token of stationary noise, but in this condition, the noise was modulated using the envelope of an eight-word masker string as described above (e.g., the envelope of “William Peter Kathy Bob lost ten green socks,” as spoken by a randomly selected talker). The two specific eight-word strings (and talkers) from which these modulation envelopes were drawn varied from trial to trial, and, as in the intelligible speech masking condition, they were always mutually exclusive with each other and with the target words/talker. Figure 2 depicts sample target/masker waveforms for each condition.

Whereas the onset of the target relative to the onset of the maskers varied based on the specific two-syllable names chosen during a given trial, the average target onset time was 2115 ms after the onset of the maskers. The average length of the target sentence was 3590 ms. The offset of the target/masker stimuli was followed by a 3000-ms retention period,^3^ given that the pupil response is a signal that is relatively slow to peak (van Rij *et al.*, 2019). After this retention period, participants were presented with a series of five response GUIs, one for each word in the target sentence. The first response GUI displayed only the word “Sue,” as this was the given (cue) word; the subsequent four GUIs contained all eight possibilities for that word (Table I). Participants selected their response from each GUI using a mouse.

### E. Adaptive tracking blocks

During the adaptive tracking portion of the experiment, participants first completed a quiet (masker-free) practice block consisting of ten trials intended to familiarize them with the target sentences and trial structure of the experiment. Next, participants completed two one-up, one-down quiet adaptive tracks that estimate the 50% correct point on the psychometric function (Levitt, 1971) designed to measure speech reception thresholds (SRTs) for these sentences and ensure adequate audibility for the remainder of the experiment. Throughout the adaptive tracking blocks, participants were instructed to maintain the same seating position to ensure consistent spatial presentation of stimuli relative to the head.

After completing these preliminary blocks, participants completed three experimental blocks in each of the three masking conditions, with block order counterbalanced across participants. In an adaptation of Brand and Kollmeier's (2002) approach, each 30-trial block consisted of two distinct but randomly interleaved 15-trial tracks designed to estimate the target-to-masker ratios (TMRs) corresponding to two different points on the psychometric function: the TMR at which the participant was predicted to achieve 75% correct intelligibility (at the word level) and the TMR at which the participant was predicted to achieve 25% correct intelligibility (also at the word level). Two different points were measured to fit the entire function and obtain a slope. Note that the first word, *Sue*, was always given and, therefore, never scored. Target sentences were always played at 40 dB sound pressure level (SPL); masker sentence levels were varied to achieve the required TMR for each trial. The target level of 40 dB was selected to balance audibility with comfort, as the tracks required frequent presentation of louder masker levels to achieve negative TMRs.

### F. EEG/pupillometry data acquisition

Participants completed the simultaneous EEG/pupillometry recording during a single study visit, which occurred on a separate day following the completion of the adaptive tracking blocks. During the EEG-pupillometry session, participants completed two blocks in each condition,^4^ with block order counterbalanced across participants. The TMR used for each condition was equal to the participant's overall 75% correct estimate in that condition (obtained by averaging the last three TMRs of each of the three adaptive tracks designed to estimate the 75% correct point and then averaging the results of those averages) and was kept constant throughout the block. Please see Table II for the TMRs at which the stimuli were presented during the physiological recording session for each participant in each condition.

**TABLE II.** TMRs during physiological recording session.

	Intelligible speech	Modulated noise	Unmodulated noise
P1	−12.15	−12.58	−8.81
P2	−8.56	−11.05	−6.47
P3	−14.68	−10.97	−7.44
P4	−15.20	−13.33	−9.18
P5	−15.14	−11.61	−7.01
P6	−17.16	−12.97	−7.59
P7	−17.19	−11.76	−8.53
P8	−12.36	−13.00	−7.85
P9	−9.86	−10.69	−13.66
P10	−15.44	−14.54	−8.87
P11	−16.10	−10.62	−9.94
P12	−7.01	−11.36	−4.74
P13	−15.39	−15.19	−7.74
P14	−11.64	−12.33	−7.20
P15	−5.37	−9.33	−7.39
*Mean*	*−12.88*	*−12.09*	*−8.16*
*s.d.* ^a^	*3.73*	*1.56*	*1.96*

^a^ Standard deviation (s.d.).

An SR Research (Ottawa, Canada) Eyelink 1000 was used during this session to record changes in pupil diameter (sampling rate: 500 Hz). Additionally, a Biosemi (Amsterdam, Netherlands) ActiveTwo system was used to acquire EEG data (sampling rate: 2048 Hz), using 32 scalp channels configured in the standard 10/20 montage, as well as four additional external facial electrodes placed to monitor eye movements and two electrodes placed on the mastoids for reference. A stationary chinrest was used to stabilize head position.

At the start of each block, the participant completed an eye gaze calibration procedure. Prior to each trial, a drift check was performed to ensure that the participant's gaze was still directed toward the center of the screen. Following the drift check, the participant clicked the mouse to start the trial. The trial began with a short period of silence, which ranged from approximately 500 to 1500 ms and was randomly jittered from trial to trial. From this point onward, the trial structure was identical to that of the adaptive tracking blocks. Participants were instructed to fixate at a circle at the center of the screen while listening. Participants were encouraged to take seated rest breaks between blocks as needed. In addition to the EEG and pupillometry data, accuracy data were collected during this session.

## III. DATA ANALYSIS

### A. Pupillometry preprocessing and data analysis

Pupil diameter measurements, in arbitrary units (AU) measured by the Eyelink 1000, were preprocessed using the R package GazeR (Geller *et al.*, 2020). Only measurements for the right pupil were included in the analysis. Initial preprocessing steps for each participant included identification and extension of blinks (100 ms pre- and post-blink), interpolation of data during and surrounding blink periods, and smoothing of the pupil trace (using a five-point moving average). Next, trials where 20% or more of the data were missing were removed from further analysis. Of the total trials analyzed, an average of 2.4% per participant were excluded during this step; this total comprised 2.8% of the intelligible speech trials, 1.9% of the modulated noise trials, and 2.5% of the unmodulated noise masking trials. Next, to identify and exclude outlier samples that may have resulted from inaccurate measurements, a histogram of all raw pupil sizes for a given participant was visually examined, and upper and lower cutoff points were determined and applied.

A subtractive baseline correction was performed for each trial. The median value of the last 500 ms of the masker-only, pre-target listening portion of each trial was used as a baseline (Mathôt *et al.*, 2018). Following baseline correction, a median absolute deviation analysis, which removes additional outliers by identifying rapid temporal changes in pupil size, was performed. Finally, baseline-corrected samples were averaged into time bins of 100 ms.

**FIG. 3.**
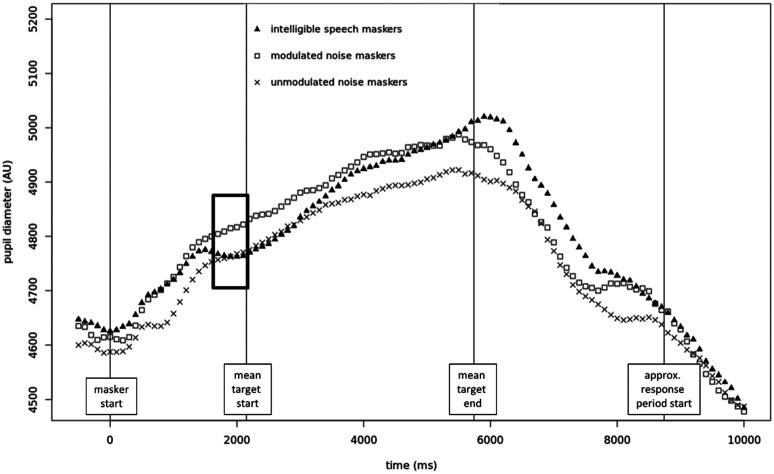
Uncorrected pupil traces in each condition, beginning 500 ms prior to the onset of the maskers. The thick black box indicates the approximate baseline period.

Because the pupil response could conceivably have differed solely depending on masker type during the baseline periods (i.e., depending on whether that baseline had consisted of unmodulated noise, modulated noise, or speech), it was determined that the raw pupil size values (i.e., the values after the data had been preprocessed and outliers removed, but prior to performing the baseline correction) should be examined to determine how any difference in baseline values/trajectories between conditions might have affected the results detailed above (see Fig. 3 for a plot of the raw pupil traces). Although the trajectories of the pupil traces during the baseline periods do appear visually different, a repeated measures analysis of variance (RM-ANOVA) found no significant effect of the masker condition on the baseline values used for baseline correction (*p* = 0.53).

### B. EEG preprocessing and data analysis

EEG data were preprocessed using EEGLAB (Delorme and Makeig, 2004), Fieldtrip (Oostenveld *et al.*, 2011), and customized matlab scripts. Initial preprocessing steps consisted of referencing to the linked mastoids, downsampling to 256 Hz, and bandpass-filtering from 1 to 30 Hz. Next, an independent component analysis was performed for each participant to identify and remove artifact components related to blinks, saccades, or noise. A blink component was identified and removed for each participant, and a saccade component was identified and removed for 14 of the 15 participants. Any remaining noisy channels were then removed and recalculated via spherical interpolation of the neighboring channels implemented in EEGLAB. Six participants required interpolation of one or more channels (among this subset of participants, an average of two channels were removed and interpolated). Following interpolation, the continuous data were epoched into individual trials, using an extended trial period from –2 to 13 s relative to the onset of the maskers. Any trials where the voltage range exceeded 200 *μ*V between 0 and 10 s relative to masker onset were excluded from further analysis. Of the total EEG trials analyzed, approximately 7.5% of the total trials were excluded during this step (this total comprised 6.4% of the intelligible speech trials, 8.6% of the modulated noise trials, and 7.5% of the unmodulated noise masking trials).

All subsequent analyses were performed using Fieldtrip and customized matlab scripts. Time-frequency representation of each trial was calculated by convolving the single-trial data using a Hanning taper (100-ms time window) from 1 to 30 Hz at a 1-Hz frequency resolution throughout the epoch for every 100 ms. An oscillatory power estimate of each trial was baseline-corrected as the relative power change from the average power estimate during baseline across all conditions for each participant. The baseline interval was set to the last 500 ms of silence immediately preceding the onset of the maskers. Finally, single-trial oscillatory power estimates were expressed as the percent change in oscillatory power for each time window and frequency, relative to the baseline interval.

Because our main focus was on the changes in the oscillatory power in the alpha (∼10 Hz) frequency range, we further inspected individual participants' baseline-corrected average time-frequency representations. One participant (P5) had an unusually elevated level of oscillatory power (at some points nearly 200 times higher than any of the other participants) across a wide band of frequencies (including part of the alpha range) just prior to, and extending into, the baseline period in the intelligible speech masking condition. No reason for this anomaly could be determined; however, it was deemed possible that it could have been artifactual. This participant's data were, therefore, removed from further analysis, yielding EEG results from *N* = 14 participants.

## IV. RESULTS

### A. Psychometric functions estimated from adaptive tracking blocks

**FIG. 4.**
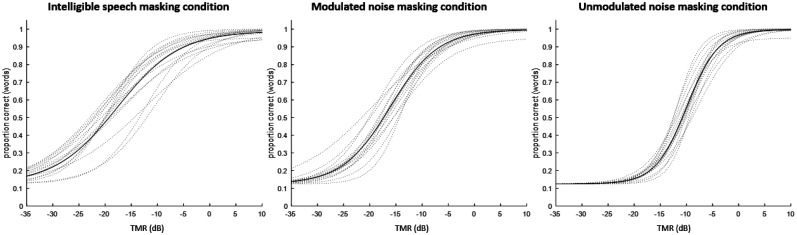
TMR-performance psychometric functions for each condition. Dotted lines indicate functions for individual participants; solid lines indicate overall functions using means of individual participants' model parameters.

To visualize the relationship between TMR and percent accuracy, a psychometric function was calculated for each participant in each condition, using the estimates (obtained during adaptive tracking) of the TMRs corresponding to the 25% correct point and 75% correct point on the psychometric function. The fitting parameters of these individual functions were then averaged by condition, and an overall function was generated for each condition, using the averaged parameters for that condition (see Fig. 4 for individual as well as overall functions). The slopes of the overall functions at midpoint were as follows: +4.4% correct per dB TMR for the intelligible speech masking condition, +5.7% correct per dB TMR for the modulated noise masking condition, and +8.6% correct per dB TMR for the unmodulated noise masking condition. The TMRs corresponding to the 50% correct points on the overall functions are as follows: –19.26 dB TMR for the intelligible speech masking condition, –17.37 dB TMR for the modulated noise masking condition, and –10.80 dB TMR for the unmodulated noise masking condition.

### B. Accuracy during EEG/pupillometry recording session

Performance accuracy during the physiological recording session was calculated at the word level for each participant to assess whether performance during this session was close to 75% (i.e., the point on the psychometric function that had been estimated during the adaptive tracking sessions and used for stimulus presentation during the physiological recording session). The mean accuracy across participants was 72.2% for the intelligible speech condition (s.d. = 5.4%), 70.8% for the modulated noise condition (s.d. = 9.6%), and 73.2% (s.d. = 7.3%) for the unmodulated noise masking condition. A RM-ANOVA comparing percent correct between the three conditions showed no significant difference: *F*(2,28) = 0.818, *p* = 0.452.

### C. Error analysis

**FIG. 5.**
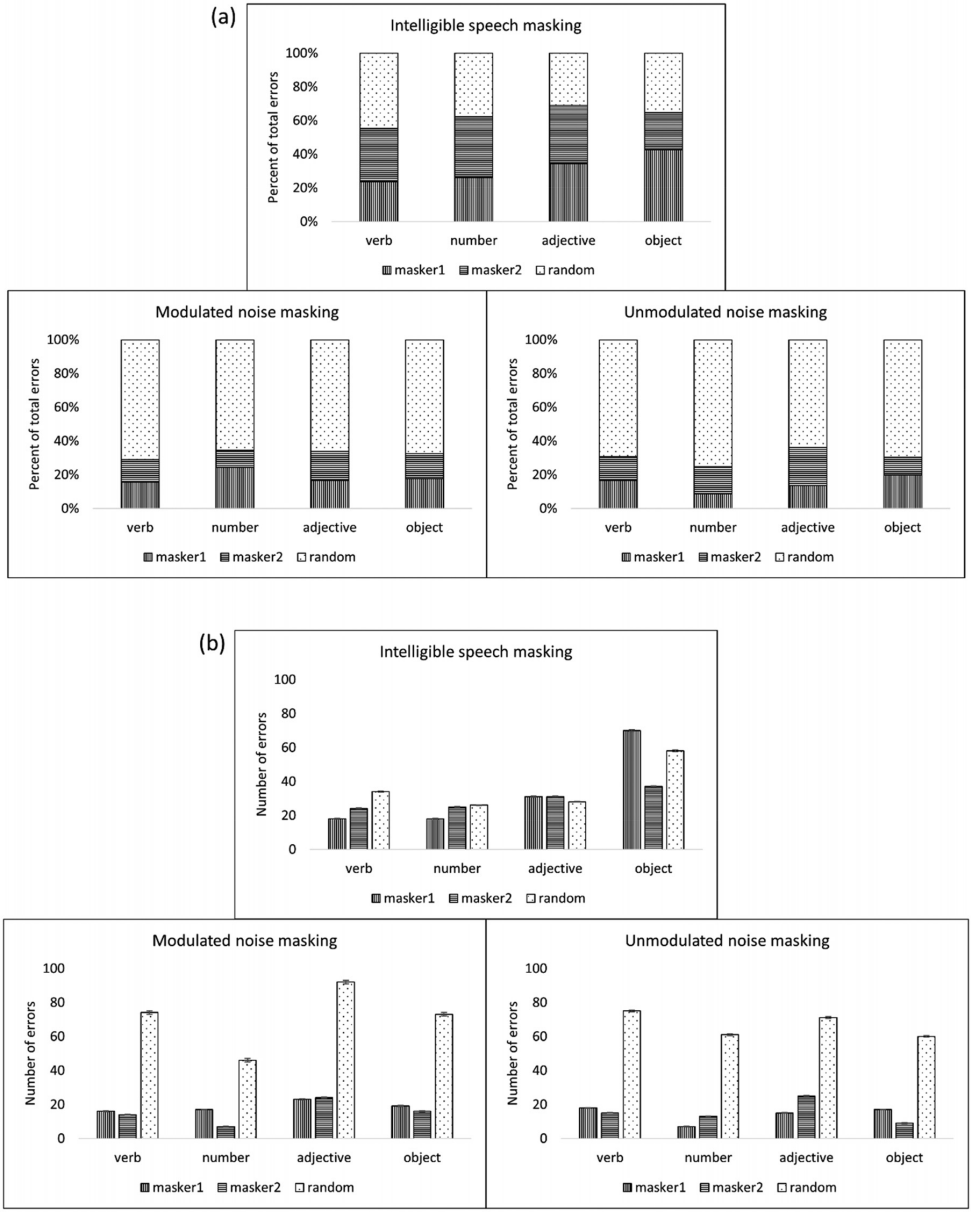
(a) Percent error type for each syntactic word in each condition. (b) Numbers of error types for each syntactic word in each condition. Error bars indicate ±1 standard error.

An analysis of error type during the physiological recording session was also performed. Figures 5(a) and 5(b) illustrate the proportions and numbers of different error types for each scored word type (verbs, numbers, adjectives, and objects) in each condition, across all participants. Within the intelligible speech masking condition, 63.5% of errors (across all word types) were masker confusion errors (i.e., the word chosen by the participant matched one of the presented masker words for that trial), whereas 36.5% were non-masker (“random”) errors. Within the modulated noise condition, 32.3% of errors (across all word types) were masker confusion errors, whereas 67.7% were random errors. Within the unmodulated noise condition, 30.8% of errors (across all word types) were masker confusion errors, whereas 69.2% were non-masker errors. Note that the masker words in the modulated noise masking condition were the words whose envelopes were applied to the noise, whereas the masker words used in this analysis for the unmodulated noise masking condition were “dummy” words that were selected during each trial but not applied to the masker stimuli in any way (i.e., if they were chosen by the participant, it was by random chance).

### D. Pupillometry results

#### 1. Effect of masking conditions on change in pupil size

**FIG. 6.**
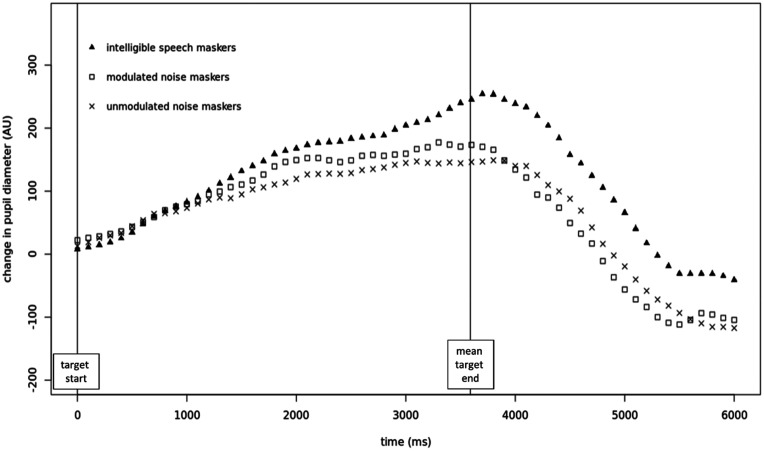
Baseline-corrected pupil traces in each condition. Mean target length = 3558 ms; s.d. = 269 ms.

Figure 6 depicts the baseline-corrected and time-binned time courses of the pupil traces for each condition, averaged across participants. Based on visual examination of this figure, the time window from 0 to 5500 ms relative to target onset was selected for all subsequent analyses of these data. This window captures the entire presentation of the target stimulus as well as the peak and drop-off of the mean pupil trace, ending as the trace begins to level off again.

Three outcome measures were calculated for each participant in each condition, using the baseline-corrected and time-binned data points within the selected time window: (1) mean pupil size, (2) peak pupil size, and (3) latency to peak. Although each of these outcome measures represents a somewhat different facet of the pupil trace, they should not be considered completely independent of one another; indeed, within each condition, they were all significantly positively correlated with one another.

**FIG. 7.**
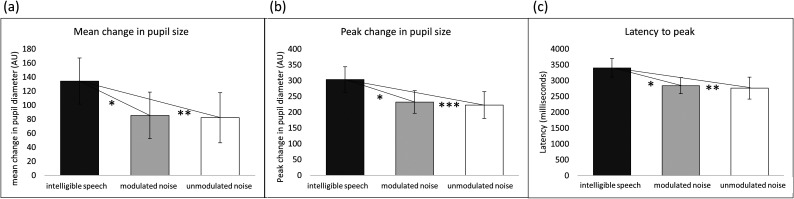
(a) Mean change in pupil size, compared across conditions. Error bars, ±1 standard error. (b) Peak change in pupil size, compared across conditions. Error bars, ±1 standard error. (c) Latency to peak, compared across conditions. Error bars, ±1 standard error.

For each of the three outcome measures, a RM-ANOVA was performed to assess the effect of masking condition on that outcome measure [see Figs. 7(a)–7(c)]. The RM-ANOVA on mean pupil size showed a significant effect of masking condition, *F*(2,28) = 4.55, *p* < 0.05, with *post hoc* testing revealing significantly higher pupil size in the intelligible speech masking condition compared to both noise masking conditions (unmodulated noise: *p* < 0.01; modulated noise: *p* < 0.05), whereas the two noise conditions did not differ from each other (*p* = 0.88).

The RM-ANOVA examining the effect of condition on peak pupil size also produced a significant result, *F*(2,28) = 7.79, *p* < 0.01, with *post hoc* testing again revealing significant differences between the intelligible speech masking condition and the unmodulated noise masking condition (*p* < 0.001), as well as between the intelligible speech masking condition and the modulated noise masking condition (*p* < 0.05), but not between the modulated and unmodulated noise masking conditions (*p* = 0.71).

Finally, the RM-ANOVA examining the effect of condition on latency to peak produced a significant result, *F*(2,28) = 4.27, *p* < 0.05, with *post hoc* testing again revealing significant differences between the intelligible speech masking condition and the unmodulated noise masking condition (*p* < 0.01), as well as between the intelligible speech masking condition and the modulated noise masking condition (*p* < 0.05), but not between the modulated and unmodulated noise masking conditions (*p* = 0.77).

#### 2. Pupil outcomes vs TMRs

**FIG. 8.**
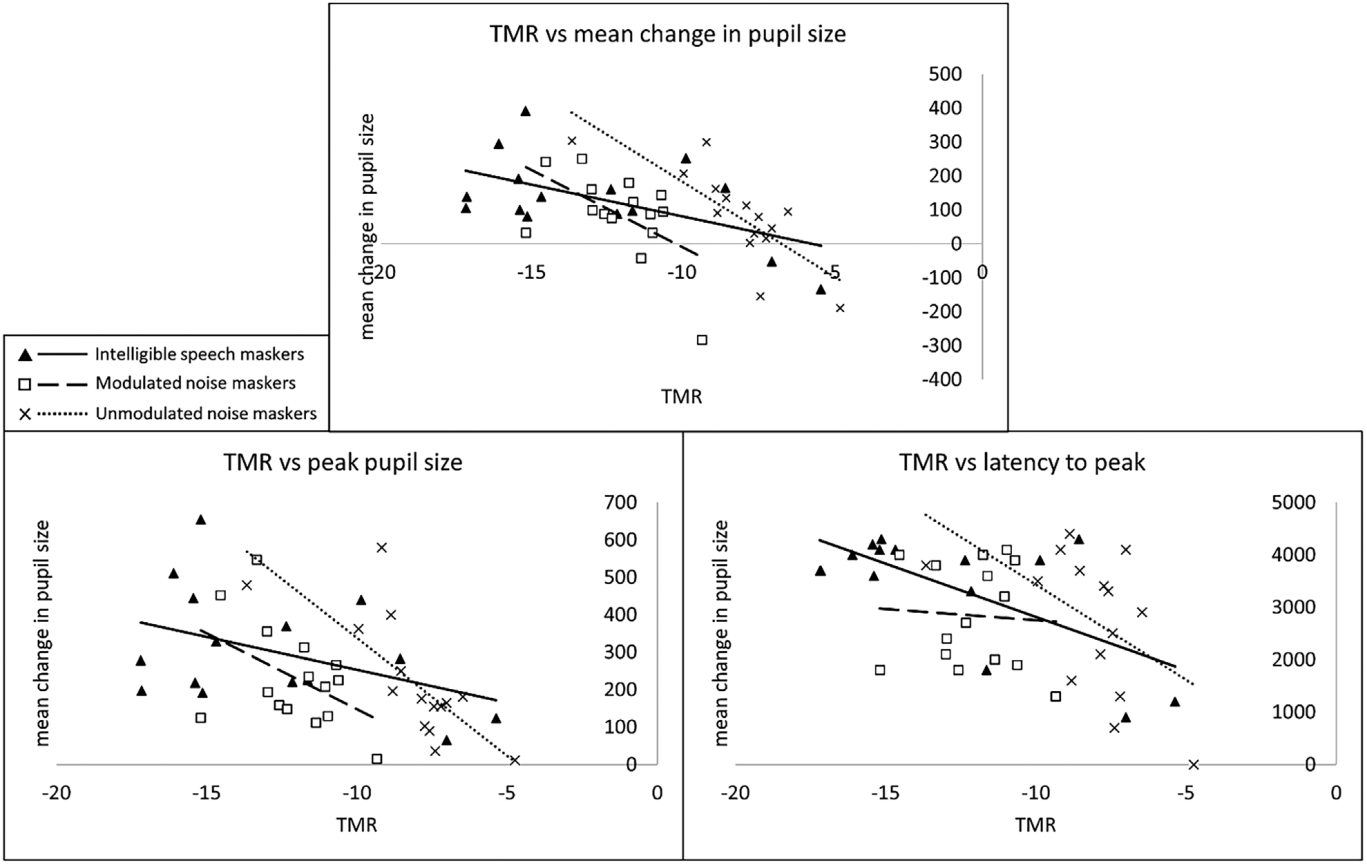
Relationships between TMR and each pupil outcome measure, in each condition.

Next, an exploratory set of Pearson correlation analyses was performed to determine whether there were significant associations between the TMRs presented during the physiological recording sessions and the three pupillometry outcome measures (mean, peak, and latency to peak). Because each participant had a different TMR for each of the three conditions, nine Pearson correlations were performed in total (Table III; Fig. 8). Of these nine correlations, six were significant (no adjustments were made for multiple comparisons).

**TABLE III.** Results of Pearson correlations between TMR and pupillometry outcomes. Values examined were within the time window of 0–5500 ms following target start.

	Mean change in pupil size	Peak change in pupil size	Latency to peak
Intelligible speech	*r* = −0.55, *p* < 0.05*	*r* = −0.42, *p* = 0.12	*r* = −0.67, *p* < 0.01**
Modulated noise	*r* = −0.55, *p* < 0.05*	*r* = −0.46, *p* = 0.09	*r* = −0.07, *p* = 0.82
Unmodulated noise	*r* = −0.79, *p* < 0.001***	*r* = −0.76, *p* < 0.01**	*r* = −0.53, *p* < 0.05*

#### 3. Growth curve analysis

All of the analyses described thus far reduced the pupil trace to a summary value (e.g., peak, mean). Although this approach captures general information about the magnitude of the pupil response and allows for easy comparison between conditions, it provides little information about how the pupil response changes over time within the window of interest. Therefore, growth curve analysis (GCA) (Mirman, 2014) was applied to better understand the overall shape and time course of the pupil response during the same period. GCA analyses were performed using the lme4 package (Bates *et al.*, 2014) in R (R Core Team, 2013).

Three sets of polynomial terms (linear, quadratic, and cubic) were included in the model to capture the pattern of each participant's mean (across-trial) pupil response time course in each condition. The masking condition was included as a fixed factor in the model, using reverse Helmert contrasts. A fixed effect for each of the three polynomial terms (poly1, poly2, and poly3) was also included, as well as fixed interaction effects between condition and each of the three polynomial terms. The model also included the random slopes and intercepts of the three polynomial terms by participant. The final model in R syntax was expressed as

pupil size∼1+poly1∗condition+poly2∗condition+poly3∗condition+(1+poly1+poly2+poly3|participant).

**FIG. 9.**
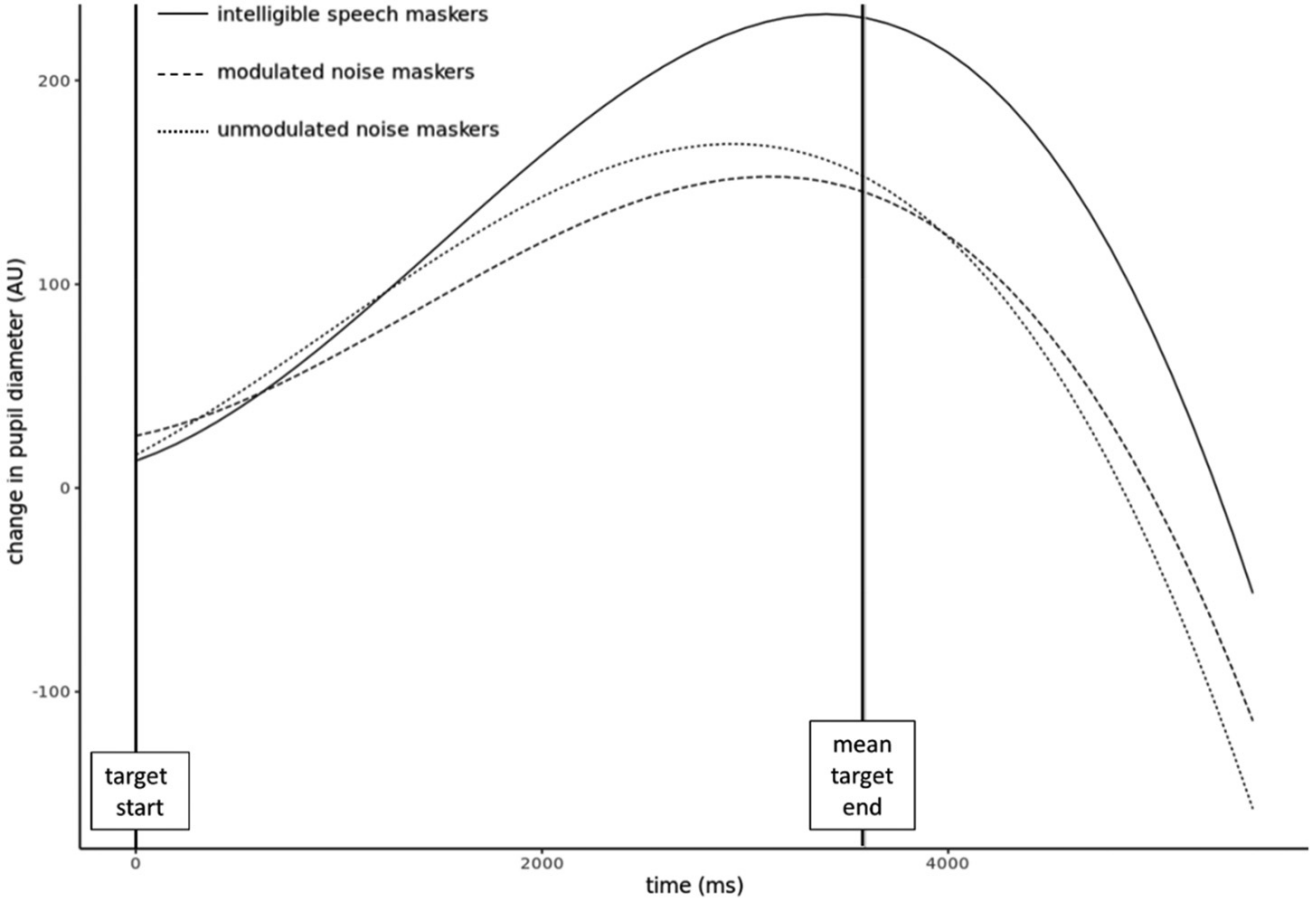
Polynomial model fit of the pupil trace in each condition.

The model's Akaike information criterion (AIC) was 28 642. Please see Fig. 9 for a plot of the model fit for each condition and Table IV for fixed effects results. One key takeaway from this analysis is that the growth of the pupil trace associated with the intelligible speech masking condition was significantly different from the pupil traces associated with the noise masking conditions (see “Factor2” in Table IV). Another is that the second and third polynomial terms (corresponding to the change in slope of the pupil trace after the peak and the change in slope just after target onset) contribute significantly to the differing shapes of the pupil traces in the different conditions.

**TABLE IV.** Fixed effects results of GCA with the intelligible speech masking condition as the reference condition, using reverse Helmert coding, conditions ordered as follows: (1) unmodulated noise maskers, (2) modulated noise maskers, (3) intelligible speech maskers. Significant effects are denoted in bold.

	Estimate	Standard error	df^a^	*t*-value	Significance
**(Intercept)**	**100.5239**	**30.8596**	**15**	**3.257**	**<0.01**
Factor1^b^	1.6835	1.6549	2460	1.017	0.309
**Factor2** ^c^	**16.8560**	**0.9554**	**2460**	**17.642**	**<0.001**
poly1	−43.2519	149.1077	15	−0.290	0.776
**poly2**	**−512.6362**	**50.2481**	**15**	**−10.202**	**<0.001**
**poly3**	**−167.6486**	**41.8759**	**15**	**−4.003**	**<0.01**
**poly1: Factor1** ^b^	**−43.5871**	**12.3839**	**2460**	**−3.520**	**<0.001**
**poly1: Factor2** ^c^	**92.3346**	**7.1498**	**2460**	**−12.914**	**<0.001**
**poly2: Factor1** ^b^	**−52.3239**	**12.3839**	**2460**	**−4.225**	**<0.001**
**poly2: Factor2** ^c^	**−14.4205**	**7.1498**	**2460**	**−2.017**	**<0.05**
poly3: Factor1^b^	4.5410	12.3839	2460	0.367	0.714
**poly3: Factor2** ^c^	**−17.9502**	**7.1498**	**2460**	**−2.511**	**<0.05**

^a^ Degrees of freedom (df).

^b^ Factor1 = modulated noise masking condition vs unmodulated noise masking condition.

^c^ Factor2 = intelligible speech masking condition vs the average of the modulated and unmodulated noise masking conditions.

### E. EEG results

#### 1. Effect of masking condition on change in alpha power

**FIG. 10.**
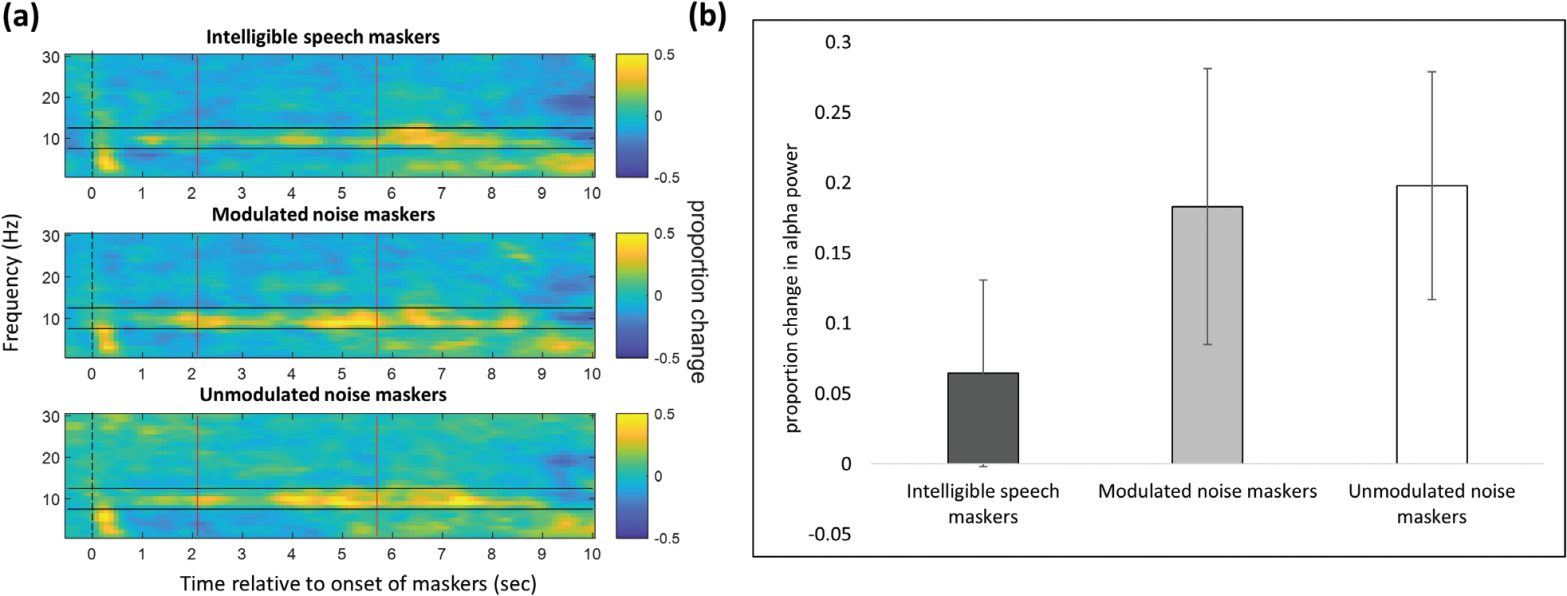
EEG data. (a) Baseline-corrected power in each condition by time-frequency unit. The dotted black line indicates the onset of the maskers, and the approximate target presentation period is bounded by vertical red lines. The alpha frequency region (8–12 Hz) is bounded by horizontal black lines. (b) Baseline-corrected power averaged across the target presentation period and across all alpha frequency units (all channels included). Error bars indicate ±1 standard error.

Figure 10 illustrates neural oscillatory power time course of each condition averaged across the 14 participants, across all channels. Note that in contrast to the pupillometry data, the baseline used for the EEG data was the 500 ms of silence immediately preceding the onset of the maskers. Our main question was to examine how alpha oscillatory power changed while listeners processed target speech under different masker conditions. Thus, we focused on the time window 2.1–5.8 s after the onset of maskers, as this window roughly captured the presentation of the target sentence (although the exact onset of the target sentence, relative to the onset of the maskers, varied somewhat from trial to trial, as did the precise length of the target sentence). We quantified average alpha power (8–12 Hz) during the time window of interest for each participant in each condition, across all scalp channels.

To statistically compare alpha power estimates between the three conditions within this window, a RM-ANOVA was performed on these overall alpha power estimates. Results of the RM-ANOVA revealed a significant main effect of masking condition, *F*(2,26) = 4.53, *p* < 0.05. *Post hoc* pairwise comparisons showed a significant difference between the intelligible speech masking condition and the unmodulated noise masking condition (*p* < 0.01), but not between the intelligible speech masking condition and the modulated noise masking condition (*p* = 0.06) or between the modulated noise masking condition and the unmodulated noise masking condition (*p* = 0.75) [see Fig. 10(b)].

#### 2. Alpha oscillatory power vs TMRs

Three Pearson correlations were performed to identify whether there were significant associations between overall alpha power and TMRs. None of these correlations revealed a significant association. Results are as follows: intelligible speech masking condition: *r* = 0.168, *p* = 0.566; modulated noise masking condition: *r* = –0.284, *p* = 0.325; unmodulated noise masking condition: *r* = 0.286, *p* = 0.322.

#### 3. Relationship between pupil response and alpha power estimates

Finally, correlational analyses were performed to determine whether there were any associations between listening effort as measured by change in pupil size and listening effort as measured by alpha power. The 13 participants whose pupillometry and EEG data were both included in the analyses detailed above were included in this analysis. To bypass differences in units and overall magnitude, the mean alpha power during the target listening period [i.e., the values included in the bar graph in Fig. 10(b)], across all participants and conditions (39 data points in total), were transformed into z-scores. Likewise, mean pupil sizes during the target listening period [i.e., the values included in the bar graph in Fig. 6(a)], across all participants and conditions (39 data points in total), were transformed into z-scores. Three Pearson correlations were then performed on these z-scores, one in each condition. None of the correlations returned a significant result (intelligible speech masking: *r* = –0.182, *p* = 0.534; modulated noise masking: *r* = –0.001, *p* = 0.996; unmodulated noise masking: *r* = –0.224, *p* = 0.442).

## V. DISCUSSION

Whereas it is known that listeners must exert cognitive effort to process speech under adverse listening conditions, it is unknown whether different types of adverse listening task demands might elicit different types of listening effort. This study, therefore, examined the effect of masker type on listening effort in young, normal-hearing individuals, measuring both changes in pupil dilations and alpha oscillatory power while listeners processed target speech under three types of masking conditions: intelligible speech maskers; speech spectrum-shaped, speech-envelope-modulated noise maskers; and speech spectrum-shaped, unmodulated noise maskers. The intelligible speech masking condition contained a high degree of IM (i.e., masking due to uncertainty), whereas the two noise masking conditions both contained a high degree of EM (i.e., masking due to spectrotemporal overlap of target and masker energy). The inclusion of both speech-envelope-modulated and unmodulated noise masking conditions allowed for an examination of the effect of the fluctuations of the broadband speech envelope on listening effort. When accuracy was held constant across the three masking conditions, we found that the intelligible speech masking condition elicited increased pupil dilation, compared to either of the noise masking conditions, but that the noise masking conditions (particularly the unmodulated noise) elicited *greater changes in alpha oscillatory power* than the intelligible speech masking condition. These findings are consistent with the view that listening effort is a multidimensional construct and that different types of auditory masking recruit distinct cognitive processes, which in turn are associated with different responses in pupil dilation and alpha-band brain oscillations.

Before discussing the pupillometry and EEG results from the current study, it is important to note that these measurements were dependent upon the TMRs used during the physiological recording session. Previous work has shown that the degree of effort required to solve a speech intelligibility task cannot be deduced from behavioral accuracy, but instead must be measured directly (Winn and Teece, 2021). To compare physiological measurements of effort across different listening conditions, therefore, the current study aimed to hold the performance level constant (at 75% word-level accuracy) across conditions using individually measured TMRs. Actual performance levels during the pupillometry/EEG recording session were indeed closely aligned with the target performance level of 75% correct in each condition (which has not always been the case when using pre-estimated TMRs; see Miles *et al.*, 2017). This approach resulted in a behavioral equivalency between conditions, supporting the conclusion that any differences observed between conditions within the physiological dataset would be indicative of true differences in listening effort.

The TMRs at which the stimuli were presented during the pupillometry/EEG recording sessions align closely with those measured in previous studies using nearly identical stimuli. Kidd *et al.* (2016) generated psychometric functions using the same five-word matrix-style target and masker sentences used here and derived an overall estimated 75% correct point between –12 and 13 dB TMR; the mean of the 75% correct estimates used during EEG/pupillometry recording for the intelligible speech masking condition in the current study was –13.1 dB TMR. Additionally, previous work has shown that high-IM listening conditions tend to elicit high variability between listeners, as well as shallower psychometric functions (Oberfeld and Kloeckner-Nowotny, 2016; Swaminathan *et al.*, 2015); as detailed in Sec. IV, the slope of the intelligible speech masking function was the shallowest of the three and had the greatest variability at midpoint. The similarity of the TMRs derived and used in the current study to those from previous work provides a level comparability between studies, despite the methodological differences, such as the use here of a different type of TMR estimation algorithm (Brand and Kollmeier, 2002), as well as the use of spatially separated loudspeakers for stimulus delivery instead of spatializing the stimuli with head-related impulse responses presented through earphones as in past work.

It should be noted that although the intelligible speech masking TMRs in the current study [as well as in Kidd *et al.* (2016)] were quite low due to the use of spatial separation between target and masker and resulting availability of binaural cues, we still considered this to be a high-IM condition for two reasons. First, previously published analysis demonstrated that, for these stimuli, more than 50% of the target energy was still available even at these low TMRs (Kidd *et al.*, 2016); second, the error analysis revealed a high percentage of masker errors in the intelligible speech masking condition, indicating substantial target-masker confusion. Another important note about the TMRs is that, whereas most participants achieved their highest (poorest) TMR in the unmodulated noise masking condition, there was otherwise substantial variability across participants regarding which of the remaining two conditions elicited the highest TMRs at the 75% correct point. Finally, previous work has provided evidence that spatially separated intelligible speech maskers do provide substantial IM: Kidd *et al.* (2019) used ideal time-frequency segregation (ITFS; see Brungart *et al.*, 2006) to remove IM from a spatially separated speech masking condition and found that this provided a 10 dB TMR difference in threshold, relative to the same condition without ITFS applied [see also Kidd and Conroy (2023)].

The key pupillometry finding of the current study was that the intelligible speech masking condition elicited significantly greater changes in pupil size than either of the noise masking conditions. This finding held true for two outcome measures related to magnitude of the pupil response relative to baseline (mean change in pupil size and peak change in pupil size), as well as for latency to peak, which was significantly later in the intelligible speech masking condition than in either of the noise conditions. All three outcome measures were strongly correlated with one another. These results are in line with the first study hypothesis and suggest that listening conditions involving highly confusable competing speech stimuli may require, or elicit, more effort on the part of the listener than listening conditions involving continuous unintelligible noise stimuli, at comparable performance levels. The polynomial model fit to the data during GCA provided additional support for differences in the shapes of the pupil traces, in particular, that the shape of the intelligible speech masking condition differed significantly in several ways from the shapes of the two noise masking conditions.

Results of the analysis of error type confirm that performance on the intelligible speech task was driven in no small part by confusion between target and masker. Errors matching one of the two masker words (as opposed to a “random” error matching neither masker word) occurred about twice as frequently in the intelligible speech masking condition (63% of total errors across all word types) vs in either of the noise masking conditions (32% and 30% of total errors across all word types in the modulated and unmodulated noise masking conditions, respectively). This disproportionately high number of masker errors in the intelligible speech masking condition demonstrates that even at a relatively high performance level (75% correct), the masker words were highly distracting to listeners. Interestingly, Winn and Teece (2021) posit that listening effort (as measured by change in pupil size) may be related to the types of errors made by the listener, as well as by the listener's own awareness and retroactive mental repair of those errors. If this is the case, it is possible that the uncertainty involved in the intelligible speech masking condition, and the much higher rate of masker confusion errors that that condition elicited (see the error analysis in Fig. 7), was related to the greater change in pupil size observed in this condition.

In addition to the significant differences between conditions, the pupillometry results also provided evidence of a relationship between TMR and pupil size, such that lower (more challenging) TMRs were generally associated with a greater change in pupil size relative to baseline. Although the reason for this association may not be apparent from this dataset, one possible interpretation is that a causal relationship exists between effort and TMR, that is, that some participants simply exerted more effort—i.e., *tried harder*—than others throughout the experiment. Listening effort during challenging tasks is voluntarily exerted and is closely tied to motivation [Dimitrijevic *et al.*, 2017; Zhang *et al.*, 2019; see Francis and Love (2020) for a review on the relationships between effort and affect/motivation], and different research participants may be more motivated or engaged than other listeners, for unknown and/or unquantifiable reasons. Under this interpretation, a hypothetical highly motivated listener might exert a high degree of effort during the adaptive tracking blocks, resulting in low TMR estimates corresponding to 75% correct. The stimuli during the pupillometry/EEG recording would then have been played at these lower TMRs, resulting (for this same highly motivated listener) in a greater change in pupil size. In contrast, a hypothetical unmotivated listener who was willing to comply with study procedures but not motivated to excel at the task might achieve higher (poorer) TMRs and, correspondingly, display lower changes in pupil size. Although this interpretation is at this point speculative, it could—if proven correct—help shed light on the listener-to-listener variability in performance so often seen in speech intelligibility tasks, particularly those containing a high degree of IM.

An alternative (or additional) explanation for the observed listener-to-listener variability is that the abilities of study participants may have varied in ways that were not related to listening effort *per se*; for example, some listeners may simply have had a greater natural ability to understand masked speech than others and/or more experience listening to masked speech. The topic of what exactly is being referred to as “listening effort”—and how quantitative measurements of this construct are affected not only by factors such as listeners' motivation and individual cognitive demands, but also by differences in their intrinsic abilities—is one that remains of considerable interest to researchers in the field. There is some evidence that performance on speech-on-speech masking tasks, especially in spatially separated conditions, may be predicted by cognitive abilities as inferred from tests such as reverse digit recall (e.g., Clayton *et al.*, 2016) and matrix-reasoning/completion tasks (e.g., Whiteford, 2023).

One final note about the pupillometry results is that the shapes of the pupil traces during the baseline period appeared to differ somewhat between conditions. Because the durations of the masker and target words varied from trial to trial, and because the pupil response is known to be relatively slow, it is difficult to draw conclusions about these differences; however, it is worth noting that, if a longer or shorter baseline period had been selected [as in Villard *et al.* (2021)], this could have impacted findings to some degree.

In contrast to the pupillometry results, the EEG results indicated a greater increase in alpha power, relative to baseline, in the unmodulated noise masking condition than in the intelligible speech masking condition during the target listening period. The pupil size and alpha power findings in the current study, therefore, displayed two different, nearly opposite patterns with regard to the degree of effort elicited by high-EM vs high-IM listening conditions. The lack of correlation between these two sets of results is in keeping with previous studies that also have found no association between different indices of listening effort (Alhanbali *et al.*, 2019; McMahon *et al.*, 2016; Miles *et al.*, 2017) and have posited that listening effort is in fact a complex construct encompassing multiple facets (Alhanbali *et al.*, 2019). Additionally, studies examining the neural substrates of EM vs IM using other approaches have found strong evidence that these different types of masking recruit different brain networks (e.g., Scott *et al.*, 2004; Szalárdy *et al.*, 2019) and may affect evoked potentials differently (e.g., Yang *et al.*, 2022). The current study's results, therefore, are consistent with this existing literature and build upon it by providing a direct comparison between the effects of high-EM vs high-IM listening tasks on change in pupil size vs change in alpha power.

The results of the current study may allow us to draw further tentative conclusions about the ways in which pupil response and EEG differ with regard to different types of masking. One interpretation of these findings is that these two physiological indices each reflect a particular facet of effort (which may be associated with specific task demands). For example, we might conclude that pupil response is indicative of the effort required to suppress a highly salient masker or to overcome target-masker uncertainty (two of the main challenges of high-IM tasks), whereas alpha power is indicative of the effort required to reconstruct and enhance the target (one of the main challenges of high-EM tasks). Because EM is the result of peripheral overlap between target and masker, it is not frequently thought of as a highly cognitively taxing task *per se* (in other words, if some of the target information is not available, then it simply is not available, and no amount of cognitive processing will change this). However, later-stage processing of target speech under challenging high-EM conditions likely requires operations such as phonemic restoration, memory template matching, and, critically, working memory, as the listener is obliged to hold pieces of the target in mind while continuing to collect additional target information so as to “fill in the gaps” created by EM. This interpretation would be consistent with previous work demonstrating that alpha power often increases during listening tasks with a high working memory load (e.g., Obleser *et al.*, 2012; Petersen *et al.*, 2015; Tuladhar *et al.*, 2007; Wöstmann *et al.*, 2017) and, in particular, a high verbal working memory load (Pavlov and Kotchoubey, 2022). Whereas better performance on high-IM tasks has been shown to be associated with good working memory (e.g., Clayton *et al.*, 2016; Koelewijn *et al.*, 2012b), high-IM tasks likely also engage additional processes necessary for differentiating between target and masker and suppressing the intelligible masker. However, more research is needed to better understand the specific challenges associated with high-EM vs high-IM tasks task demands, as well as how these demands contribute to effortful listening.

## VI. CONCLUSION

This study examined the effect of masker type on listening effort, as indexed by both pupillometry and EEG. Results showed that pupil size and alpha power, while both considered indices of listening effort, responded differently to high-IM vs high-EM listening tasks—specifically, that pupil size increased more in response to a high-IM task, whereas alpha increased more in response to a high-EM task. These results add to the current understanding of the role of effort in listeners in everyday communicative situations and help shed light on the relationship between two widely used physiological indices of listening effort. It is hoped that results from this study may be used in future work aimed at more precisely measuring effort under different types of listening conditions and may also help inform the selection of physiological indices to assess listening effort. Gaining a full understanding of listening effort as a complex construct will result in more accurate quantification of the degree of effort elicited by specific listening tasks and situations, as well as the effect of the exertion of effort on the listener and on task performance.

## Footnotes

^1^
Races of participants were self-reported. “Multiracial” indicates that the participant reported two or more races.

^2^
This study was approved and overseen by the Boston University Institutional Review Board.

^3^
One participant (P1) completed the adaptive tracks with a retention period of 6000 ms; this period was deemed to be unnecessarily long and was shortened to 3000 ms for all subsequent participants. All participants (including P1) completed the EEG/pupillometry recording session with a retention period of 3000 ms.

^4^
For P8, data from one of the modulated noise masking conditions were accidentally overwritten. This participant returned to redo this block on a different day; however, for logistical reasons, only pupillometry data were able to be recorded during this redo session. Therefore, P8 only contributed one modulated noise masking block to the EEG data.

## References

[1] Alhanbali, S. , Dawes, P. , Millman, R. E. , and Munro, K. J. (2019). “ Measures of listening effort are multidimensional,” Ear Hear. 40(5), 1084–1097. 10.1097/AUD.0000000000000697 30747742 PMC7664710

[2] Bates, D. , Mächler, M. , Bolker, B. , and Walker, S. (2014). “ Fitting linear mixed-effects models using lme4,” arXiv:1406.5823.

[3] Brand, T. , and Kollmeier, B. (2002). “ Efficient adaptive procedures for threshold and concurrent slope estimates for psychophysics and speech intelligibility tests,” J. Acoust. Soc. Am. 111(6), 2801–2810. 10.1121/1.1479152 12083215

[4] Brungart, D. S. , Chang, P. S. , Simpson, B. D. , and Wang, D. (2006). “ Isolating the energetic component of speech-on-speech masking with ideal time-frequency segregation,” J. Acoust. Soc. Am. 120(6), 4007–4018. 10.1121/1.2363929 17225427

[5] Brungart, D. S. , Simpson, B. D. , Ericson, M. A. , and Scott, K. R. (2001). “ Informational and energetic masking effects in the perception of multiple simultaneous talkers,” J. Acoust. Soc. Am. 110(5), 2527–2538. 10.1121/1.1408946 11757942

[6] Cherry, E. C. (1953). “ Some experiments on the recognition of speech, with one and with two ears,” J. Acoust. Soc. Am. 25(5), 975–979. 10.1121/1.1907229

[7] Clayton, K. K. , Swaminathan, J. , Yazdanbakhsh, A. , Zuk, J. , Patel, A. D. , and Kidd, G., Jr. (2016). “ Executive function, visual attention and the cocktail party problem in musicians and non-musicians,” PLoS One 11(7), e0157638. 10.1371/journal.pone.0157638 27384330 PMC4934907

[8] Delorme, A. , and Makeig, S. (2004). “ EEGLAB: An open source toolbox for analysis of single-trial EEG dynamics including independent component analysis,” J. Neurosci. Methods 134, 9–21. 10.1016/j.jneumeth.2003.10.009 15102499

[9] Dimitrijevic, A. , Smith, M. L. , Kadis, D. S. , and Moore, D. R. (2017). “ Cortical alpha oscillations predict speech intelligibility,” Front. Hum. Neurosci. 11, 88. 10.3389/fnhum.2017.00088 28286478 PMC5323373

[10] Dimitrijevic, A. , Smith, M. L. , Kadis, D. S. , and Moore, D. R. (2019). “ Neural indices of listening effort in noisy environments,” Sci. Rep. 9(1), 11278. 10.1038/s41598-019-47643-1 31375712 PMC6677804

[11] Fiedler, L. , Ala, T. S. , Graversen, C. , Alickovic, E. , Lunner, T. , and Wendt, D. (2021). “ Hearing aid noise reduction lowers the sustained listening effort during continuous speech in noise—A combined pupillometry and EEG study,” Ear Hear. 42(6), 1590–1601. 10.1097/AUD.0000000000001050 33950865

[12] Francis, A. L. , and Love, J. (2020). “ Listening effort: Are we measuring cognition or affect, or both?” Wiley Interdiscip. Rev. Cogn. Sci. 11(1), e1514. 10.1002/wcs.1514 31381275

[13] Geller, J. , Winn, M. B. , Mahr, T. , and Mirman, D. (2020). “ GazeR: A package for processing gaze position and pupil size data,” Behav. Res. 52(5), 2232–2255. 10.3758/s13428-020-01374-8 PMC7544668 32291732

[14] Gilzenrat, M. S. , Nieuwenhuis, S. , Jepma, M. , and Cohen, J. D. (2010). “ Pupil diameter tracks changes in control state predicted by the adaptive gain theory of locus coeruleus function,” Cogn. Affect. Behav. Neurosci. 10(2), 252–269. 10.3758/CABN.10.2.252 20498349 PMC3403821

[15] Haro, S. , Rao, H. M. , Quatieri, T. F. , and Smalt, C. J. (2022). “ EEG alpha and pupil diameter reflect endogenous auditory attention switching and listening effort,” Eur. J. Neurosci. 55(5), 1262–1277. 10.1111/ejn.15616 35098604 PMC9305413

[16] Joshi, S. , Li, Y. , Kalwani, R. M. , and Gold, J. I. (2016). “ Relationships between pupil diameter and neuronal activity in the locus coeruleus, colliculi, and cingulate cortex,” Neuron 89(1), 221–234. 10.1016/j.neuron.2015.11.028 26711118 PMC4707070

[17] Kidd, G., Jr. , and Colburn, H. S. (2017). “ Informational masking in speech recognition,” in *The Auditory System at the Cocktail Party*, edited by J. C. Middlebrooks , J. Z. Simon , A. N. Popper , and R. R. Fay ( Springer, New York), pp. 75–109. 10.1007/978-3-319-51662-2_4

[18] Kidd, G., Jr. , and Conroy, C. (2023). “ Auditory informational masking,” Acoust. Today 19(1), 29–36. 10.1121/AT.2023.19.1.29

[19] Kidd, G., Jr. , Mason, C. R. , Best, V. , Roverud, E. , Swaminathan, J. , Jennings, T. , Clayton, K. , and Colburn, H. S. (2019). “ Determining the energetic and informational components of speech-on-speech masking in listeners with sensorineural hearing loss,” J. Acoust. Soc. Am. 145(1), 440–457. 10.1121/1.5087555 30710924 PMC6347574

[20] Kidd, G., Jr. , Mason, C. R. , Richards, V. M. , Gallun, F. J. , and Durlach, N. I. (2008). “ Informational masking,” in *Auditory Perception of Sound Sources*, edited by W. A. Yost , A. N. Popper , and R. R. Fay ( Springer, New York), pp. 143–190. 10.1007/978-0-387-71305-2_6

[21] Kidd, G., Jr. , Mason, C. R. , Swaminathan, J. , Roverud, E. , Clayton, K. K. , and Best, V. (2016). “ Determining the energetic and informational components of speech-on-speech masking,” J. Acoust. Soc. Am. 140(1), 132–144. 10.1121/1.4954748 27475139 PMC5392100

[22] Koelewijn, T. , Zekveld, A. A. , Festen, J. M. , and Kramer, S. E. (2012a). “ Pupil dilation uncovers extra listening effort in the presence of a single-talker masker,” Ear Hear. 33(2), 291–300. 10.1097/AUD.0b013e3182310019 21921797

[23] Koelewijn, T. , Zekveld, A. A. , Festen, J. M. , Rönnberg, J. , and Kramer, S. E. (2012b). “ Processing load induced by informational masking is related to linguistic abilities,” Int. J. Otolaryngol. 2012, 865731. 10.1155/2012/865731 23091495 PMC3471442

[24] Levitt, H. C. C. H. (1971). “ Transformed up‐down methods in psychoacoustics,” J. Acoust. Soc. Am. 49(2B), 467–477. 10.1121/1.1912375 5541744

[25] Mathôt, S. , Fabius, J. , Van Heusden, E. , and Van der Stigchel, S. (2018). “ Safe and sensible preprocessing and baseline correction of pupil-size data,” Behav. Res. 50(1), 94–106. 10.3758/s13428-017-1007-2 PMC5809553 29330763

[26] Mattys, S. L. , Davis, M. H. , Bradlow, A. R. , and Scott, S. K. (2012). “ Speech recognition in adverse conditions: A review,” Lang. Cogn. Process. 27, 953–978. 10.1080/01690965.2012.705006

[27] McMahon, C. M. , Boisvert, I. , de Lissa, P. , Granger, L. , Ibrahim, R. , Lo, C. Y. , Miles, K. , and Graham, P. L. (2016). “ Monitoring alpha oscillations and pupil dilation across a performance-intensity function,” Front. Psychol. 7, 745. 10.3389/fpsyg.2016.00745 27252671 PMC4877370

[28] Miles, K. , McMahon, C. , Boisvert, I. , Ibrahim, R. , De Lissa, P. , Graham, P. , and Lyxell, B. (2017). “ Objective assessment of listening effort: Coregistration of pupillometry and EEG,” Trends Hear. 21, 2331216517706396. 10.1177/2331216517706396 28752807 PMC5536372

[29] Mirman, D. (2014). *Growth Curve Analysis and Visualization Using R* (CRC Press, Boca Raton, FL).

[30] Oberfeld, D. , and Kloeckner-Nowotny, F. (2016). “ Individual differences in selective attention predict speech identification at a cocktail party,” eLife 5, e16747. 10.7554/eLife.16747 27580272 PMC5441891

[31] Obleser, J. , and Weisz, N. (2012). “ Suppressed alpha oscillations predict intelligibility of speech and its acoustic details,” Cereb. Cortex 22(11), 2466–2477. 10.1093/cercor/bhr325 22100354 PMC4705336

[32] Obleser, J. , Wöstmann, M. , Hellbernd, N. , Wilsch, A. , and Maess, B. (2012). “ Adverse listening conditions and memory load drive a common alpha oscillatory network,” J. Neurosci. 32(36), 12376–12383. 10.1523/JNEUROSCI.4908-11.2012 22956828 PMC6621258

[33] Ohlenforst, B. , Wendt, D. , Kramer, S. E. , Naylor, G. , Zekveld, A. A. , and Lunner, T. (2018). “ Impact of SNR, masker type and noise reduction processing on sentence recognition performance and listening effort as indicated by the pupil dilation response,” Hear. Res. 365, 90–99. 10.1016/j.heares.2018.05.003 29779607

[34] Oostenveld, R. , Fries, P. , Maris, E. , and Schoffelen, J. M. (2011). “ FieldTrip: Open source software for advanced analysis of MEG, EEG, and invasive electrophysiological data,” Comput. Intell. Neurosci. 2011, 156869. 10.1155/2011/156869 21253357 PMC3021840

[35] Pavlov, Y. G. , and Kotchoubey, B. (2022). “ Oscillatory brain activity and maintenance of verbal and visual working memory: A systematic review,” Psychophysiology 59(5), e13735. 10.1111/psyp.13735 33278030

[36] Peelle, J. E. (2018). “ Listening effort: How the cognitive consequences of acoustic challenge are reflected in brain and behavior,” Ear Hear. 39(2), 204–214. 10.1097/AUD.0000000000000494 28938250 PMC5821557

[37] Petersen, E. B. , Wöstmann, M. , Obleser, J. , Stenfelt, S. , and Lunner, T. (2015). “ Hearing loss impacts neural alpha oscillations under adverse listening conditions,” Front. Psychol. 6, 177. 10.3389/fpsyg.2015.00177 25745410 PMC4333793

[38] R Core Team (2013). *R: A Language and Environment for Statistical Computing* ( R Foundation for Statistical Computing, Vienna, Austria).

[39] Rennies, J. , Best, V. , Roverud, E. , and Kidd, G., Jr. (2019). “ Energetic and informational components of speech-on-speech masking in binaural speech intelligibility and listening effort,” Trends Hear. 23, 2331216519854597. 10.1177/2331216519854597 31172880 PMC6557024

[40] Rennies, J. , Schepker, H. , Holube, I. , and Kollmeier, B. (2014). “ Listening effort and speech intelligibility in listening situations affected by noise and reverberation,” J. Acoust. Soc. Am. 136, 2642–2653. 10.1121/1.4897398 25373965

[41] Sauseng, P. , and Klimesch, W. (2008). “ What does phase information of oscillatory brain activity tell us about cognitive processes?” Neurosci. Biobehav. Rev. 32(5), 1001–1013. 10.1016/j.neubiorev.2008.03.014 18499256

[42] Scott, S. K. , Rosen, S. , Wickham, L. , and Wise, R. J. (2004). “ A positron emission tomography study of the neural basis of informational and energetic masking effects in speech perception,” J. Acoust. Soc. Am. 115(2), 813–821. 10.1121/1.1639336 15000192

[43] Seifi Ala, T. , Graversen, C. , Wendt, D. , Alickovic, E. , Whitmer, W. M. , and Lunner, T. (2020). “ An exploratory study of EEG alpha oscillation and pupil dilation in hearing-aid users during effortful listening to continuous speech,” PLoS One 15(7), e0235782. 10.1371/journal.pone.0235782 32649733 PMC7351195

[44] Swaminathan, J. , Mason, C. R. , Streeter, T. M. , Best, V. , Kidd, G., Jr. , and Patel, A. D. (2015). “ Musical training, individual differences and the cocktail party problem,” Sci. Rep. 5(1), 11628. 10.1038/srep11628 26112910 PMC4481518

[45] Szalárdy, O. , Tóth, B. , Farkas, D. , György, E. , and Winkler, I. (2019). “ Neuronal correlates of informational and energetic masking in the human brain in a multi-talker situation,” Front. Psychol. 10, 786. 10.3389/fpsyg.2019.00786 31024409 PMC6465330

[46] Tuladhar, A. M. , Huurne, N. T. , Schoffelen, J. M. , Maris, E. , Oostenveld, R. , and Jensen, O. (2007). “ Parieto‐occipital sources account for the increase in alpha activity with working memory load,” Hum. Brain Mapp. 28(8), 785–792. 10.1002/hbm.20306 17266103 PMC6871495

[47] van der Wel, P. , and van Steenbergen, H. (2018). “ Pupil dilation as an index of effort in cognitive control tasks: A review,” Psychon. Bull. Rev. 25(6), 2005–2015. 10.3758/s13423-018-1432-y 29435963 PMC6267528

[48] van Rij, J. , Hendriks, P. , van Rijn, H. , Baayen, R. H. , and Wood, S. N. (2019). “ Analyzing the time course of pupillometric data,” Trends Hear. 23, 2331216519832483. 10.1177/2331216519832483 31081486 PMC6535748

[49] Versfeld, N. J. , Lie, S. , Kramer, S. E. , and Zekveld, A. A. (2021). “ Informational masking with speech-on-speech intelligibility: Pupil response and time-course of learning,” J. Acoust. Soc. Am. 149(4), 2353–2366. 10.1121/10.0003952 33940918

[50] Villard, S. , Perrachione, T. , Lim, S. J. , Alam, A. , and Kidd, G. (2021). “ Listening effort elicited by energetic versus informational masking,” Proc. Mtgs. Acoust. 45, 050002. 10.1121/2.0001546

[51] Visentin, C. , Valzolgher, C. , Pellegatti, M. , Potente, P. , Pavani, F. , and Prodi, N. (2022). “ A comparison of simultaneously-obtained measures of listening effort: Pupil dilation, verbal response time and self-rating,” Int. J. Audiol. 61, 561–573. 10.1080/14992027.2021.1921290 34634214

[52] Wendt, D. , Koelewijn, T. , Książek, P. , Kramer, S. E. , and Lunner, T. (2018). “ Toward a more comprehensive understanding of the impact of masker type and signal-to-noise ratio on the pupillary response while performing a speech-in-noise test,” Hear. Res. 369, 67–78. 10.1016/j.heares.2018.05.006 29858121

[53] Whiteford, K. (2023). “ Association of musical training with auditory and speech neural coding and perception,” in *Proceedings of the 46th Midwinter Research Meeting*, Brentwood, TN ( Association for Research in Otolaryngology, Brentwood, TN), Vol. 46, p. 99.

[54] Winn, M. B. , and Teece, K. H. (2021). “ Listening effort is not the same as speech intelligibility score,” Trends Hear. 25, 23312165211027688. 10.1177/23312165211027688 34261392 PMC8287270

[55] Winn, M. B. , Wendt, D. , Koelewijn, T. , and Kuchinsky, S. E. (2018). “ Best practices and advice for using pupillometry to measure listening effort: An introduction for those who want to get started,” Trends Hear. 22, 2331216518800869. 10.1177/2331216518800869 30261825 PMC6166306

[56] Wisniewski, M. G. , Zakrzewski, A. C. , Bell, D. R. , and Wheeler, M. (2021). “ EEG power spectral dynamics associated with listening in adverse conditions,” Psychophysiology 58(9), e13877. 10.1111/psyp.13877 34161612 PMC8355203

[57] Wöstmann, M. , Lim, S. J. , and Obleser, J. (2017). “ The human neural alpha response to speech is a proxy of attentional control,” Cereb. Cortex 27(6), 3307–3317. 10.1093/cercor/bhx074 28334352

[58] Yang, X. , Liu, L. , Yang, P. , Ding, Y. , Wang, C. , and Li, L. (2022). “ The effects of attention on the syllable-induced prepulse inhibition of the startle reflex and cortical EEG responses against energetic or informational masking in humans,” Brain Sci. 12(5), 660. 10.3390/brainsci12050660 35625046 PMC9139428

[59] Zekveld, A. A. , Koelewijn, T. , and Kramer, S. E. (2018). “ The pupil dilation response to auditory stimuli: Current state of knowledge,” Trends Hear. 22, 2331216518777174. 10.1177/2331216518777174 30249172 PMC6156203

[60] Zekveld, A. A. , Kramer, S. E. , and Festen, J. M. (2010). “ Pupil response as an indication of effortful listening: The influence of sentence intelligibility,” Ear Hear. 31(4), 480–490. 10.1097/AUD.0b013e3181d4f251 20588118

[61] Zhang, M. , Siegle, G. J. , McNeil, M. R. , Pratt, S. R. , and Palmer, C. (2019). “ The role of reward and task demand in value-based strategic allocation of auditory comprehension effort,” Hear. Res. 381, 107775. 10.1016/j.heares.2019.107775 31401432

